# An In Situ Reflectance Spectroscopic Investigation to Monitor Two-Dimensional MoS_2_ Flakes on a Sapphire Substrate

**DOI:** 10.3390/ma13245794

**Published:** 2020-12-18

**Authors:** Yina Wang, Lei Zhang, Wen Yang, Shanshan Lv, Chenhui Su, Hang Xiao, Faye Zhang, Qingmei Sui, Lei Jia, Mingshun Jiang

**Affiliations:** School of Control Science and Engineering, Shandong University, Jingshi Road, Jinan 250061, China; ynwangsdu@163.com (Y.W.); wenyang0@126.com (W.Y.); sdulvshanshan@163.com (S.L.); suchenhui2010@163.com (C.S.); 15275139781@163.com (H.X.); zhangfaye@sdu.edu.cn (F.Z.); sdusqm@126.com (Q.S.); sdujial@126.com (L.J.); sdujiangmingshun@163.com (M.J.)

**Keywords:** differential reflectance spectroscopy (DRS), in situ, molybdenum disulfide (MoS_2_), sapphire substrate, chemical vapor deposition (CVD)

## Abstract

In this work, we demonstrate the application of differential reflectance spectroscopy (DRS) to monitor the growth of molybdenum disulfide (MoS_2_) using chemical vapor deposition (CVD). The growth process, optical properties, and structure evolution of MoS_2_ were recorded by in-situ DRS. Indeed, blue shifts of the characteristic peak B were discussed with the decrease of temperature. We also obtained the imaginary part of the MoS_2_ dielectric constant according to reflectance spectra. This method provides an approach for studying the change of two-dimensional (2D) materials’ dielectric constant with temperature. More importantly, our work emphasizes that the DRS technique is a non-destructive and effective method for in-situ monitoring the growth of 2D materials, which is helpful in guiding the preparation of 2D materials.

## 1. Introduction

Transition metal dichalcogenides (TMDCs) have attracted unprecedented attention in the last few years. As layered thin-film materials, two-dimensional (2D) TMDCs have massive advantages of the adjustable band gap, high carrier mobility, and excellent mechanical properties [1,2,3,4]. Molybdenum disulfide (MoS_2_) is one of the most popular 2D TMDCs materials. The monolayer MoS_2_ has a direct band-gap, which is between graphene with zero band-gap and hexagonal boron nitride with wide band-gap. It is considered to have attractive prospects for sensors, catalysis, supercapacitor, and optoelectronic devices [5,6,7,8,9,10,11]. It is crucial to acquire high-quality materials for industrial applications. Nevertheless, the research on the controllable preparation of high-quality materials still needs to be investigated. It is of great significance to explore the growth process and mechanisms of 2D materials for realizing the controllable preparation of thin 2D materials. Moreover, the interactions between substrates and materials can affect the structure and the growth status of the materials [12,13,14]. For instance, surface properties of substrates have an impact on the charge doping and strain for materials [14]. The transparent substrates, such as sapphire, mica, and quartz, are generally used as growth substrates of 2D materials. However, a systematic investigation of the growth mechanism on transparent substrates is still in the embryonic stage.

The in-situ observations during the growth of 2D materials have considerable advantages in analyzing the growth mechanisms. Ringe and collaborators observed in situ changes of TMDCs optical properties during electroablation using spectroelectrochemical micro-extinction spectroscopy (SE-MExS) [15]. CVD is considered to be the most potential preparation technology to obtain high-quality materials among many preparation methods for 2D materials [14,16]. Nonetheless, there are limited reported on in-situ characterizations of CVD in the published literatures [17,18,19,20]. Wang et al. introduced the in-situ observation of monolayer molybdenum disulfide synthesized on a SiO_2_/Si substrate by Raman spectroscopy and in situ images [17]. Kasırga’s group explored the process of the salt-assisted TMDCs growth through optical micrographs using the modified CVD chamber [18]. Nevertheless, most of these reports emphasized the growth variation of 2D materials on opaque substrates because of the outstanding optical contrast, especially SiO_2_/Si substrate. Sun’s team used transmission spectroscopy to report the growth of molybdenum disulfide on the sapphire substrate [19]. They studied the significance of transmission spectra characterization for online growth detection and ignored the influence of the scattering on the spectra. More importantly, this research elucidates that the optical characterization technique is a powerful tool for detecting CVD-grown 2D materials. Differential reflectance spectroscopy (DRS) is a kind of optical detection technology with high sensitivity [20,21,22]. The DRS detection system is easy to set up and can be used to characterize variations on the substrate surface. Therefore, we can utilize DRS to investigate the evolution of as-grown 2D materials on the transparent substrate.

Here, as-grown CVD MoS_2_ flakes on the transparent sapphire substrate were explored. The evolution of MoS_2_ optical performances during the growth period was monitored by the in situ DRS. It was found that the position of the characteristic peak B shifted blue with a decrease of temperature. Meanwhile, the structure of MoS_2_ flakes was also characterized by the imaginary part of the dielectric constant. Our results showed a variation of the imaginary part of the dielectric constant with temperature for MoS_2_ flakes. Additionally, optical microscopy, Raman spectra, ex-situ DRS, and atomic force microscopy (AFM) were ex-situ conducted to verify the formation of MoS2 flakes at room temperature.

## 2. Materials and Methods

### 2.1. CVD Growth of MoS_2_ Flakes on the Sapphire Substrate

The atmospheric pressure CVD (APCVD) was used to prepare the MoS_2_ flakes on the single-crystalline sapphire (0001) substrate. The sample was synthesized in a tubular furnace with two temperature zones. High purity sulfur powder (1.0 g, 99.99 wt%, Alfa Aesar, Shanghai, China) and MoO_3_ powder (15 mg, 99.99 wt%, Alfa Aesar, Shanghai, China) were supplied as two precursors for the growth of MoS_2_. The MoO_3_ power was placed on the alumina boat, which was located 6.0 cm upstream from a sapphire substrate in the second temperature zone of the tube furnace. Another alumina boat with sulfur power was put in the first temperature zone. First, the air in the reaction chamber was evacuated by a vacuum pump, and then the reaction chamber was filled with high-purity argon to the atmospheric pressure. After that, we continued to ventilate into the furnace for 5 min to remove air as much as possible. Subsequently, MoO_3_ powder was heated to 600 °C in 30 min. Afterward, it continued to be ramped to 800 °C in 30 min and maintained for 40 min. Finally, the furnace was naturally cooled down to room temperature. During the growth condition, argon gas as the carrier gas was injected into a reaction chamber to convey vapor of precursors to the down-stream substrate.

### 2.2. In Situ Characterization

**Figure 1:**
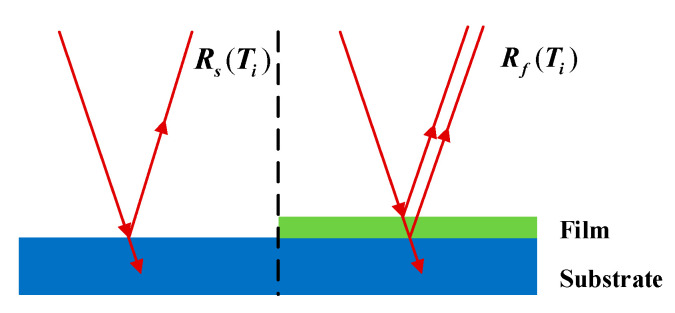
The schematic diagram of in-situ DRS model in the variable temperature environment.

For the in-situ characterization experiment, original reflected intensity data was collected by the homemade online optical system. The detailed process can be found in Reference [20]. In situ DRS technology is utilized to process the reflected intensity, and it is expressed by:

(1) $$\frac{\Delta R}{R}=\frac{R_{f}(T_{i})-R_{s}(T_{i})}{R_{s}(T_{i})},$$

where $R_{s}(T_{i})$ and $R_{f}(T_{i})$ denote the reflected intensity of the bare substrate and one of CVD grown flakes at the same temperature $T_{i}$. It is necessary to gather the reflected intensity $R_{s}(T_{i})$ of the bare substrate with the whole temperature range before the TMDCs preparation. The schematic illustration of the in-situ DRS model displays in Figure 1. By the above-mentioned data processing, in-situ DRS values can effectively suppress the influence of high temperature on growth information. This makes DRS an optical probe technique for characterizing TMDCs prepared by CVD. Additionally, this method is suitable under conditions that the gas atmosphere has no absorption properties and the detected material has optical absorption in the measurement wavelength range.

### 2.3. Ex Situ Measurements

MoS_2_ flakes prepared on the sapphire substrate were systematically off-line measured by optical microscope, Raman spectra, ex-situ DRS, and atomic force microscope (AFM). These measurements were operated at room temperature. Firstly, optical images were obtained with an optical microscope (GFM-550, Shanghai Guangmi Instrument Co., Ltd., Shanghai, China). Secondly, Raman spectra were used to evaluate the quality and structure of the sample by Raman microscope (HR Evolution 800, Horiba, Paris, France). A ×50 objective lens and the excitation wavelength of 532 nm were used. Afterward, ex-situ DRS at room temperature were obtained by $DRS=(R_{F}-R_{0})/R_{0}$, where $R_{F}$ and $R_{0}$ represent the reflected intensity of the substrate surfaces covered with and without MoS_2_ flakes, respectively. At last, the thickness of MoS_2_ flakes was acquired by an AFM (Dimension Icon, Bruker, Santa Barbara, CA, USA) in PeakForce tapping mode.

### 2.4. Calculation of Dielectric Constant

Based on the Fresnel formula, we can establish a relationship between the DRS values and the dielectric constant of the material [23]. When a substrate is transparent within the measured light range, the imaginary parts of the extinction coefficient and dielectric constant for the substrate are equal to zero. Therefore, the imaginary part $\varepsilon^{\prime \prime }$ of MoS_2_ dielectric constant can be calculated from DR values, defined as: [21,24]

(2) $$\varepsilon^{\prime \prime }=\frac{DR\cdot \left(1-n_{s}^{2}\right)\lambda }{8\pi d_{f}},$$

where $n_{s}$ is the refractive index of the substrate, λ is the wavelength of detection light, and $d_{f}$ is the average thickness of flakes on the substrate. In addition, $d_{f}$ is defined by $d_{f}=d\cdot \theta_{f}$, where d denotes the thickness of monolayer MoS_2_ and $\theta_{f}$ represents the partial coverage of the observation area on the substrate. As a result, the $\varepsilon^{\prime \prime }$ data is the characteristic of the material on the substrate with a coverage of $\theta_{f}$. Here, the thickness of the sample is acquired by AFM and the coverage is deduced by optical microscopy images.

## 3. Results and Discussion

### 3.1. In Situ Reflectance Spectroscopy

**Figure 2:**
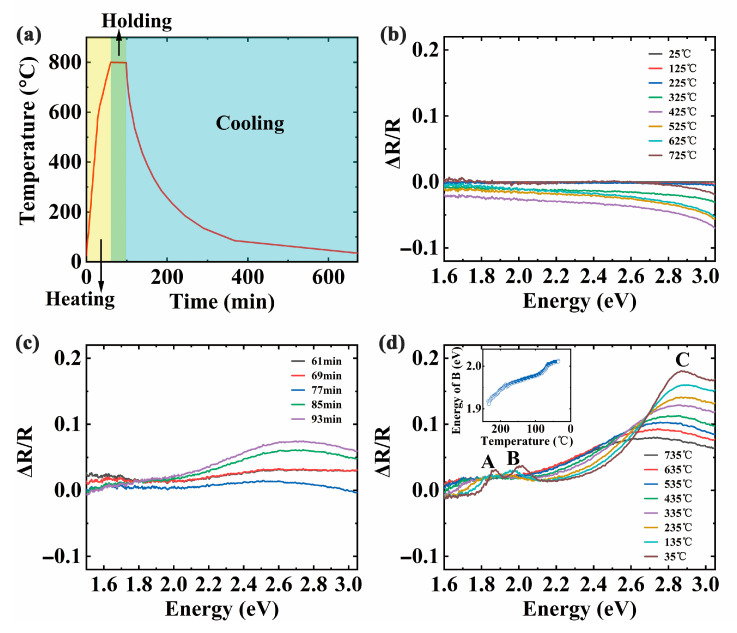
(**a**) The temperature profile during the growth of molybdenum disulfide (MoS_2_) flakes using chemical vapor deposition (CVD); (**b**–**d**) In-situ differential reflectance spectroscopy (DRS) of the heating, holding, and cooling sections during the CVD growth, respectively. The inset in (**d**) is the variation of peak B with temperature. A, B, and C represent three characteristic peaks of MoS_2_ at different energy, respectively.

We prepared MoS_2_ flakes on the sapphire substrate by CVD using MoO_3_ and sulfur as precursors. The reverse side of a sapphire substrate was roughened to avoid interference influence [25]. In order to get a deep insight into the formation of the MoS_2_ flakes, we employed the DRS technique to monitor the overall evolution during the growing MoS_2_ on the sapphire substrate. As illustrated in Figure 2a, the entire experimental process is divided into three sections by temperature variations: heating, holding, and cooling sections. With a close inspection in Figure 2b, the ∆R/R signals are plotted every 100 °C for the heating section. It is essential to point out that the optical absorption performance hardly appears in Figure 2b. One can deduce that there are almost no MoS_2_ flakes covering the substrate surface for this section, which can be attributed that the temperature of the furnace was relatively low. Thus, the spectra of the heating section display no distinct characteristic peaks of MoS_2_ flakes. Figure 2c shows the evolution of DRS signals during the holding section, in which the spectrum is drawn every 8 min. The absorption peak around 2.70 eV gradually becomes prominent with the increase of time. This peak is consistent with the MoS_2_ characteristic peak called C peak, which is related to van Hove singularity. It can be interpreted by the broad spectral transition between K and Γ in the Brillouin region, where the conduction bands are almost parallel to the valence bands [26]. Compared with other absorption peaks, the peak C is more easily observed. It could be related that van Hove singularity can enhance the interaction between light and matter and result in photon absorption enhancement [27]. Notably, the red-shift of peak C occurred due to higher temperatures compared with room temperature. Furthermore, the sudden changes of DRS intensity near 77 min and 85 min are associated with the thermal deformation of mechanical structures, such as the sample stage. In-situ DRS during the cooling section are plotted per every 100 °C in Figure 2d. There are three characteristic peaks of MoS_2_ corresponding to peak A, B, and C. As the experiment progressed, the two characteristic peaks A and B located at 1.86 eV and 2.01 eV gradually appeared respectively. They arise from the exciton transitions of K point in the Brillouin region. The energy difference between peak A and B can be attributed to the valence band separation caused by spin-orbit coupling [28]. In addition, as illustrated in the insertion graph of Figure 2d, the peak B had blue shifts with the decrease of temperature. It comes from the increased overlap of orbits that form the band during the cooling section [29]. This observation provides direct evidence that the absorption features of MoS_2_ flakes are affected by temperature.

**Figure 3:**
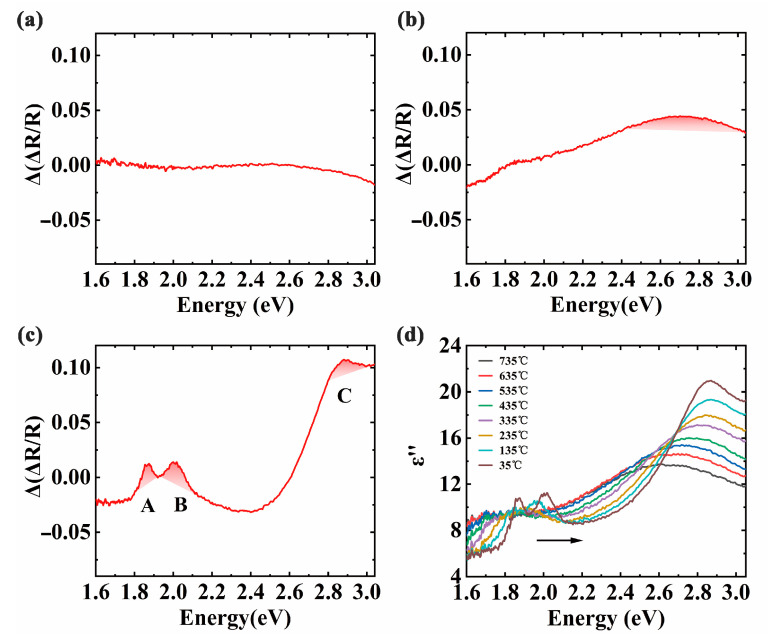
(**a**–**c**) The increments of DRS during heating, holding and cooling section for the CVD growth, respectively. A, B, and C represent three characteristic peaks of MoS_2_ at different energy, respectively; (**d**) The imaginary part values $\varepsilon^{\prime \prime }$ of MoS_2_ dielectric constant per 100 °C during the cooling section. The arrow indicates the direction of the peak as the temperature decreases.

On the basis of the investigation above, a closer inspection of the incremental DRS signals is exhibited in Figure 3. As one can see from Figure 3a, the alteration of ∆(∆R/R) spectrum is slight for the heating section. It can be speculated that MoS_2_ domains have hardly been formed. In Figure 3b, there is a prominent peak at 2.70 eV in the ∆(∆R/R) spectrum during the holding section. It can be inferred that the surface of the sapphire substrate has been covered with MoS_2_ flakes. For the cooling section sketched in Figure 3c, the increment of DRS reveals distinct peaks corresponding to the characteristic peaks A, B, and C of MoS_2_ flakes, respectively. Note that this result has also provided direct evidence for the growth of MoS_2_ flakes. Furthermore, we also calculated the dielectric constant to analyze the growth process of MoS_2_. Figure 3d shows the imaginary parts $\varepsilon^{\prime \prime }$ of the MoS_2_ dielectric constant at different temperatures. The amplitudes are slightly different compared with the literature due to different measurement methods and the effect of sample uniformity [16]. More importantly, it can be recognized that the peaks of $\varepsilon^{\prime \prime }$ also are blue-shifted with the decrease of temperature, which can be attributed to the reduced electron-phonon interaction and the decrement of lattice constant [30,31]. Consequently, the detailed growth evolution of MoS_2_ flakes can be revealed from in-situ reflection spectra and their increments.

### 3.2. Ex-Situ Characterization

**Figure 4:**
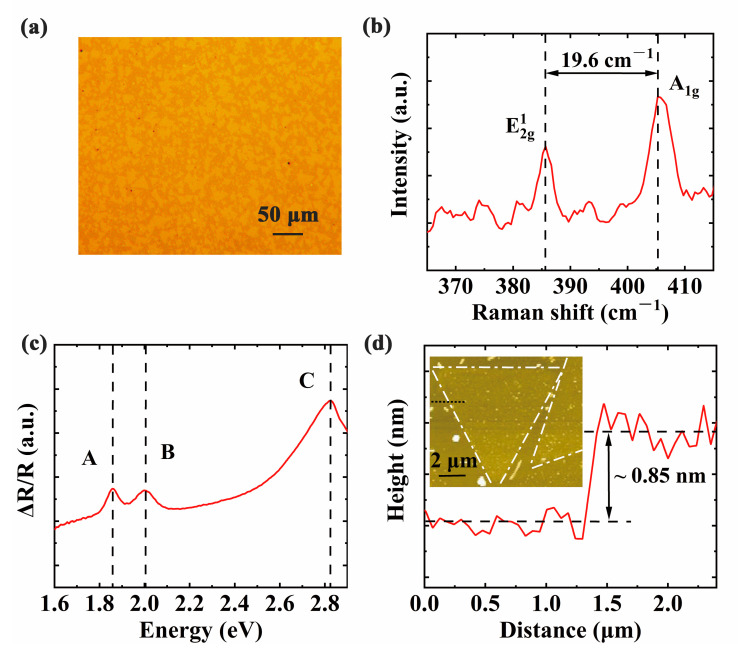
The ex-situ characterizations of the MoS_2_ sample at room temperature. (**a**) An optical image of MoS_2_ flakes on the sapphire substrate. The scale bar is 50 μm; (**b**,**c**) Raman spectra and DRS of MoS_2_ flakes respectively. A, B, and C represent three characteristic peaks of MoS_2_ at different energy, respectively.; (**d**) An atomic force microscopy (AFM) height profile recorded the triangular MoS_2_ flakes along the black short-dot line. Triangular MoS2 flakes are marked with white dash-dot lines. The inset is the corresponding graph with the scale bar of 2 μm.

To further probe the morphology and structure of the MoS_2_ flakes, optical microscopy, Raman spectroscopy, ex-situ DRS, and AFM were performed at room temperature, respectively. Figure 4a displays the optical image of the sample on the sapphire substrate, which reveals the triangular morphology of MoS_2_ flakes with flat surfaces. Their side lengths ranged from 10 to 30 μm in size. Raman characterization displays two characteristic peaks in Figure 4b. The peak E^1^_2g_ at 385.7 cm^−1^ is attributed to the in-plane vibration mode of MoS_2_, while the peak A_1g_ at 405.3 cm^−1^ is associated with the out-of-plane vibration mode. It is worth to mention that the frequency difference between the E^1^_2g_ and A_1g_ is sensitive to the layer thickness. Raman frequency difference of MoS_2_ flakes is ~19.6 cm^−1^, in good agreement with that of monolayer MoS_2_ in previous studies [32]. Thus, the thickness for the sample can be interpreted by a monolayer structure. Moreover, ex-situ DRS at room temperature is presented in Figure 4c. The absorption peaks A, B, and C of MoS_2_ domains appear at 1.86 eV, 2.01 eV, and 2.83 eV, respectively. Additionally, AFM was used to assess the thickness of as-grown MoS_2_ flakes on the sapphire. A height profile along the black broken line is about 0.85 nm in Figure 4d, which is consistent with the results of monolayer MoS_2_ thickness reported in the literature [32,33]. An AFM image in the inset of Figure 4d reveals that the sample has the classic monolayer topography. Therefore, ex-situ characterization results confirm that MoS_2_ flakes have synthesized on the sapphire substrate in accordance with that of in-situ DRS.

## 4. Conclusions

In summary, the growth process of MoS_2_ flakes synthesized by CVD on the transparent sapphire substrate was in situ monitored. We adopted reflectance spectroscopy to successfully record the evolution of MoS_2_ optical characteristics during CVD growth. Specifically, the growth of the MoS_2_ flakes began in the holding section. It is worth mentioning that the characteristic peak C has more prominent absorption performance at higher temperatures compared to the absorption peak A and B. More importantly, blue shifts of peak B were recorded with the influence of temperature. In addition, the dielectric constant of MoS_2_ was also obtained. This result can provide a method to observe the changes of dielectric constant for 2D materials with different temperatures. It is essential to point out that in situ DRS can also be applied to investigate the preparation of other 2D materials on the sapphire substrate. This work can contribute to a better understanding of the 2D materials growth mechanism and controllable synthesis.
